# Lipid Raft-Mediated Regulation of Hyaluronan–CD44 Interactions in Inflammation and Cancer

**DOI:** 10.3389/fimmu.2015.00420

**Published:** 2015-08-20

**Authors:** Toshiyuki Murai

**Affiliations:** ^1^Department of Microbiology and Immunology, Graduate School of Medicine, Osaka University, Suita, Japan

**Keywords:** extracellular matrix remodeling, oligosaccharides, cholesterol, membrane raft, membrane dynamics, transmembrane signaling, a disintegrin and metalloproteinase, ectodomain shedding

## Abstract

Hyaluronan is a major component of the extracellular matrix and plays pivotal roles in inflammation and cancer. Hyaluronan oligomers are frequently found in these pathological conditions, in which they exert their effects via association with the transmembrane receptor CD44. Lipid rafts are cholesterol- and glycosphingolipid-enriched membrane microdomains that may regulate membrane receptors while serving as platforms for transmembrane signaling at the cell surface. This article focuses on the recent discovery that lipid rafts regulate the interaction between CD44 and hyaluronan, which depends largely on hyaluronan’s size. Lipid rafts regulate CD44’s ability to bind hyaluronan in T cells, control the rolling adhesion of lymphocytes on vascular endothelial cells, and regulate hyaluronan- and CD44-mediated cancer cell migration. The implications of these findings for preventing inflammatory disorders and cancer are also discussed.

## Introduction

**Figure 1:**
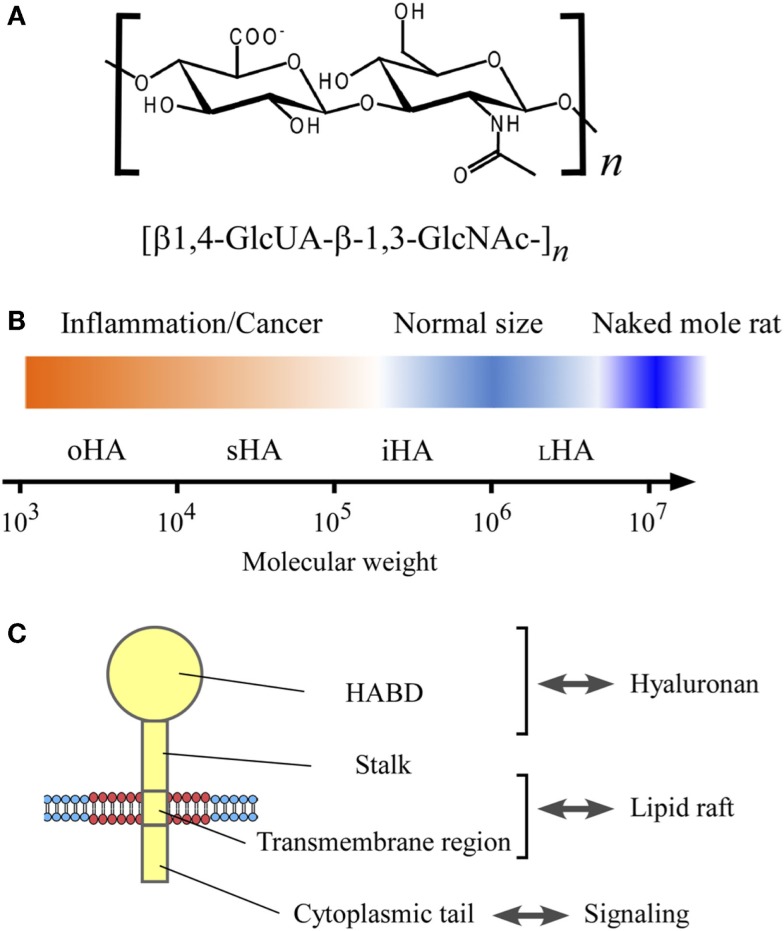
**The structure and size of hyaluronan, and its interaction with CD44 in the lipid raft**. **(A)** Chemical structure of hyaluronan. Hyaluronan is a linear glycosaminoglycan consisting of repeating disaccharide units of d-glucuronic acid (GlcUA) and *N*-acetyl-glucosamine (GlcNAc) with the structure [β1,4-GlcUA-β-1,3-GlcNAc-]*n*. **(B)** Hyaluronan size and its relevance to inflammation and cancer. The sizes of hyaluronan are categorized according to Weigel’s nomenclature (5), and shown in the log scale of the molecular weight: oligomeric hyaluronan (oHA; 1 × 10^3^–1 × 10^4^), small hyaluronan (sHA; 1 × 10^4^–1 × 10^5^), intermediate hyaluronan (iHA; 1 × 10^5^–1 × 10^6^), and large hyaluronan (_L_HA; 1 × 10^6^–1 × 10^7^). Hyaluronan’s molecular weight in normal physiological conditions ranges from 1 × 10^5^ to 1 × 10^7^, whereas the low-molecular weight hyaluronan has relevance to inflammation and cancer. The very high-molecular weight hyaluronan from naked mole rat (molecular weight, 6 × 10^6^–1.2 × 10^7^) has an anti-malignant activity (6). **(C)** CD44 structure. CD44 consists of four regions; hyaluronan-binding domain (HABD), stalk domain, transmembrane region, and cytoplasmic tail.

Hyaluronan is a linear glycosaminoglycan consisting of repeating disaccharide units of d-glucuronic acid (GlcUA) and *N*-acetyl-d-glucosamine (GlcNAc) with the structure [β1,4-GlcUA-β-1,3-GlcNAc-]*n*, and a physiological molecular weight (relative molecular mass) ranging from 1 × 10^5^ to 1 × 10^7^ with polydispersity (1) (Figure 1). Hyaluronan was first purified from bovine vitreous humor in 1934 (2). It is now known to be ubiquitous in vertebrate tissues, with particular abundance in connective tissues, such as synovial fluid, Wharton’s jelly in the umbilical cord, and the vitreous humor of the eye, where it plays a mechanical role determined by its viscous features. While hyaluronan is traditionally regarded as a space filling, structural molecule involved in lubricating joints or holding connective tissues in place (3), it also functions as a microenvironmental cue in inflammation and cancer (4).

## Hyaluronan: A Size-Dependent Bioactive Molecule

### Structure and physicochemical properties

Hyaluronan has a simple structure that lacks a core protein linkage or sulfation. It is synthesized as a large, negatively charged linear polymer with multiple carboxyl groups on GlcUA residues. Both the GlcUA and GlcNAc residues are in the β configuration, which allows their bulky groups, including the hydroxyl and carboxyl groups, to reside in sterically unhindered equatorial positions, and thus hyaluronan forms particularly stable tertiary structures in aqueous solution that exhibit remarkable hydrodynamic properties, including non-Newtonian viscosity and water retention. In dilute solution, hyaluronan forms an expanded random coil due to the mutual repulsion of its carboxyl groups, and at higher concentrations it forms an entangled meshwork, the size of which depends on its concentration and molecular weight, and on the ionic strength and pH of the solution (7). At physiological ionic strengths, hyaluronan’s polyanionic structure causes the partition and diffusion of monovalent ions, such as Na^+^ and Cl^−^ as well as the divalent cation Ca^2+^ at a nearly ideal Donnan equilibrium (8).

### Biosynthesis

While most glycosaminoglycans are synthesized in the Golgi apparatus, hyaluronan is synthesized at the cell surface, from uridine 5′-diphosphate (UDP)-GlcUA and UDP-GlcNAc by hyaluronan synthases (HASs), a class of membrane-integrated gylcosyltransferases (EC 2.4.1.212). There are three HAS isoforms in mammals, such as HAS1, HAS2, and HAS3 (9), which have different tissue- and cell-specific expression patterns and *K*_m_ values for their substrates; they also synthesize hyaluronan of different sizes *in vitro* (10). Various growth factors, including epidermal growth factor (EGF), platelet-derived growth factor (PDGF), and transforming growth factor-β (TGF-β), induce the transcription of *HAS* genes and enhance hyaluronan synthesis (11, 12). Dysregulated HAS expression or activity is sometimes associated with tissue injury and immune diseases (13).

### Degradation

Hyaluronan is enzymatically degraded mainly by hyaluronidases. The mammalian hyaluronidases (EC 3.2.1.35) are endo-β-acetyl-hexosaminidases, which hydrolyze the hexosaminidic β1,4-linkages between GlcNAc and GlcUA residues (14). Six hyaluronidase-like sequences are present in the human genome; the five genes, i.e., *HYAL1, HYAL2, HYAL3, HYAL4*, and *SPAM1* genes, which encode Hyal-1, Hyal-2, Hyal-3, Hyal-4, and PH-20, respectively, and a pseudogene *PHYAL1* that is transcribed but not translated (15). Among these isoforms, Hyal-1 and Hyal-2 are predominantly active in somatic tissues (16). Hyal-1 is an acid-active lysosomal enzyme, and catalyzes the hydrolytic degradation of hyaluronan with any molecular weight, generating predominantly tetrasaccharides (17). Hyal-2 is an acid-active glycosylphosphatidylinositol (GPI)-anchored enzyme, and digests hyaluronan polymers to products with a molecular weight of approximately 2 × 10^4^, i.e., 100 saccharides (18). In addition to enzymatic degradation, hyaluronan can be depolymerized by reactive oxygen species generated by oxidative stress (and/or reactive nitrogen species), which cause random cleavage of the endoglycosidic linkages (19).

The degradation of large hyaluronan to low-molecular weight hyaluronan occurs at sites of inflammation including atherosclerosis, rheumatoid arthritis, and tumorigenesis, and low-molecular weight hyaluronan promotes inflammation (20). Low-molecular weight hyaluronan arises by the action of hyaluronidases, and the upregulation of expression and activity of hyaluronidases have been noticed in such inflammation conditions (13). Reactive oxygen species accumulate at sites of inflammation, where low-molecular weight hyaluronan can arise also by oxidative degradation.

### Size

Hyaluronan is a major component of extracellular matrix and plays important roles in development and tissue remodeling. Under normal physiological conditions, hyaluronan has a high average molecular weight (>1 × 10^6^) and exhibits immunosuppressive effects: high-molecular weight hyaluronan suppresses septic responses to lipopolysaccharides (21), inhibits lymphocyte-mediated cytolysis (22), and has anti-angiogenic effects (23). Under pathological conditions, such as inflammation and cancer, extracellular matrix remodeling is upregulated. In this situation, the hyaluronan is more polydispersed, showing a preponderance of low-molecular weight forms (13). In general, low-molecular weight hyaluronan is highly immunostimulatory, inflammatory, and angiogenic.

Table 1 summarizes the biological activities associated with different sizes of hyaluronan. The terms used in the literature to describe hyaluronan’s sizes are confusing and inconsistent. Therefore, this article uses the system proposed by Weigel (5), in which hyaluronan’s sizes are categorized according to the log of the molecular weight: oligomeric hyaluronan (oHA; 1 × 10^3^–1 × 10^4^), small hyaluronan (sHA; 1 × 10^4^–1 × 10^5^), intermediate hyaluronan (iHA; 1 × 10^5^–1 × 10^6^), and large hyaluronan (_L_HA; 1 × 10^6^–1 × 10^7^) (Figure 1B). As shown in Table 1, low-molecular weight hyaluronans (oHA, sHA, and iHA) generally exhibit inflammation- and cancer-promoting activities (5, 6, 24–47). The other effects of low-molecular weight hyaluronan on gene expression are well summarized elsewhere (13). Notably, studies in the naked mole rat (*Heterocephalus glaber*), an extraordinarily long-lived rodent with low cancer incidence, show that while low-molecular weight hyaluronan has pro-malignant or pro-inflammatory effects, very high-molecular weight hyaluronan (6 × 10^6^–1.2 × 10^7^) has an anti-malignant activity (6) (Figure 1B). Another study shows that oligomeric hyaluronan of 6–40 saccharides, which is frequently found in tumor-bearing patients, enhances cleavage of the hyaluronan receptor CD44 in malignant tumor cells, and concomitantly upregulates CD44-dependent tumor cell migration, whereas larger polymers of hyaluronan fail to enhance CD44 cleavage and migration (24). Collectively, low-molecular weight hyaluronan tends to function as a “danger signal” (48).

**Table 1:** **Size-dependent biological activities of hyaluronan**.

Cell type	Hyaluronan size^a^	Receptor	Activity	Reference
**oHA (1** ***×*** **10^3^**–**1** ***×*** **10^4^)**				
Human glioblastoma	6, 8, 10, 12-mer, 6.9 × 10^3^	CD44	Enhance CD44 shedding and cell migration	(24)
Human ovarian carcinoma	2.5 × 10^3^ (4 ∼ 20-mer)	CD44	Inhibit the RTK–CD44 association	(25)
Human peripheral nerve sheath tumor	2.5 × 10^3^ (4 ∼ 20-mer)	CD44	Inhibit the BCRP–CD44 association	(26)
Human breast carcinoma	2.5 × 10^3^ (6 ∼ 20-mer)	CD44	Inhibit lactate influx	(27)
Rat glioma	2.5 × 10^3^ (6 ∼ 20-mer)	CD44	Suppress growth	(28)
Human prostate, colon, and breast carcinoma	2.5 × 10^3^ (6 ∼ 20-mer)	CD44	Inhibit the activation of RTKs	(29)
Human colon carcinoma	2.5 × 10^3^ (6 ∼ 20-mer)	CD44	Inhibit ErbB2 phosphorylation	(30)
Human colon, mouse mammary carcinoma	2.5 × 10^3^ (6 ∼ 20-mer)	CD44	Suppress PI3K/Akt cell survival pathway	(31)
	(8 × 10^4^, 2 × 10^6^)	–	No effect	(31)
Human breast cancer	2.5 × 10^3^ (6 ∼ 20-mer)	CD44	Abrogate CD44 clustering and stimulate ERK	(32)
Rat fibroblast	6-mer, 10-mer	CD44	Inactivate ERM	(33)
Mouse and human glioma	10-mer	CD44	Enhance hyaluronan synthesis	(34)
Mouse brain capillary EC	12-mer	CD44	Induce differentiation	(35)
Rat dermal fibroblast	6-mer, 8-mer	CD44, RHAMM	Stimulate wound repair	(36)
	(4 × 10^4^)	CD44, RHAMM	Inhibit wound closure	(36)
Bovine aortic EC	1.4 × 10^3^–4.5 × 10^3^	CD44, RHAMM	Activate PLCγ1, Src, and ERK	(37)
Human dermal microvascular EC	4–6-mer	TLR4	Increase chemokine production	(38)
Mouse Lewis lung carcinoma	4–6-mer	Unknown^b^	Induce MMP expression	(39)
	(4 × 10^6^)	–	No effect	(39)
Human dendritic cells	4–14-mer	Unknown^c^	Induce production of TNF-α, IL-1β, and IL-12	(40)
	(8 × 10^4^–2 × 10^5^, 2 × 10^5^–1 × 10^6^)	–	No effect	(40)
**sHA (1** ***×*** **10^4^**–**1** ***×*** **10^5^)**				
Human vascular SMC	2 × 10^4^–5 × 10^5^	CD44	Stimulate cell-cycle progression	(41)
	(4 × 10^6^)	CD44	Inhibit cell-cycle progression	(41)
Human cervical cancer	2.3 × 10^4^	CD44	Enhance chemokinesis	(42)
	(9.2 × 10^5^)	–	No effect	(42)
Mouse macrophage cell line	2.5 × 10^4^–7.5 × 10^4^	CD44	Facilitate GAS phagocytosis	(43)
	(8 × 10^5^–1.2 × 10^6^)	CD44	Limit GAS phagocytosis	(43)
Human colon carcinoma	3.5 × 10^4^	TLR4	Induce HβD2 expression	(44, 45)
	(4.7 × 10^3^, 2 × 10^6^)	–	No effect	(45)
HEK293 transfectant	8 × 10^4^–1.8 × 10^5^	HARE	Activate NF-κB-mediated gene expression	(5)
	(<6.6 × 10^3^, >4.4 × 10^5^)	–	No effect	(5)
**iHA (1** ***×*** **10^5^**–**1** ***×*** **10^6^)**				
Mouse macrophage cell line	4.7 × 10^5^	CD44	Induce chemokine production	(46)
	(6 × 10^6^)	–	No effect	(46)
Human primary monocyte	5 × 10^4^–6 × 10^5^, 2 × 10^5^	TLR4	Stimulate arachidonic acid release	(47)
	(4 × 10^3^, 2.5 × 10^6^)	–	No effect	(47)
**_L_HA (1** ***×*** **10^6^**–**1** ***×*** **10^7^)**				
Naked mole rat fibroblast	6 × 10^6^–1.2 × 10^7^	CD44	Transformation resistant	(6)
	(3 × 10^3^)	CD44	Transformation susceptible	(6)

*BCRP, breast cancer resistance protein/ABCG2; EC, endothelial cells; ERK, extracellular signal-regulated kinase; ERM, ezrin/radixin/moesin; GAS, group A Streptococcus; HARE, hyaluronic acid receptor for endocytosis; HβD2, human β-defensin 2; IL-12, interleukin-12; IL-1β, interleukin-1β; MMP, matrix metalloproteinase; NF-κB, nuclear factor-κB; PI3K, phosphoinositide 3-kinase; PLCγ1, phospholipase Cγ1; RHAMM, receptor for hyaluronan-mediated motility; RTK, receptor tyrosine kinase; SMC, smooth muscle cell; TLR4, Toll-like receptor 4; TNF-α, tumor necrosis factor-α*.

*^a^Molecular weight or number of saccharides (mer)*.

*^b^The effect of hyaluronan was independent of CD44, RHAMM, and TLR4 (39)*.

*^c^The effect of hyaluronan was independent of CD44 and RHAMM (40)*.

### Receptors for hyaluronan

The major cell-surface receptor for hyaluronan is CD44, a widely distributed type-I transmembrane glycoprotein that is implicated in a wide variety of biological processes, including cell adhesion and migration, as well as in inflammation and cancer (49). CD44 mediates the adhesion and dissemination of immune and cancer cells through its association with hyaluronan (50, 51) (Figure 1C). In addition to hyaluronan, CD44’s interaction with certain growth factors also plays important roles in cancer progression (52). Receptor for hyaluronan-mediated motility (RHAMM)/CD168 is another major hyaluronan receptor expressed in a variety of cell types, and it plays important roles in tissue injury and repair and in tumor cell motility (53). Other hyaluronan receptors include lymphatic vessel endothelial hyaluronan receptor 1 (LYVE-1) (54) and hyaluronan receptor for endocytosis (HARE)/stabilin-2 (55). LYVE-1 is mainly restricted to the endothelium of lymph nodes and lymphatic vessels, while HARE is expressed in sinusoidal endothelial cells of the liver, spleen, and lymph nodes, and it mediates the systemic clearance of hyaluronan from the vascular and lymphatic circulatory systems.

## Lipid Rafts

### Lipid raft structure

The plasma membrane is a dynamic mixture of proteins and lipids that forms the boundary and interface between the intracellular space and the cellular environment. The traditional model of cellular membrane structure was the fluid mosaic model proposed by Singer and Nicolson, in which globular proteins float in a lipid bilayer with an amphipathic structure (56). Later, non-homogenously distributed assemblies of lipids were found in the plasma membrane of many cell types, and the model was improved by Simons and van Meer, who suggested the existence of small domains called lipid rafts (57). In the understanding of the lipid raft model, cholesterol- and sphingolipid-enriched microdomains of the plasma membrane assumed a biophysical state resembling a liquid-ordered (*L*_o_) phase floating within a liquid-disordered (*L*_d_) membrane phase (58). In that model, the representative proteins with raft affinity were GPI-anchored proteins. The finding that GPI-anchored proteins were isolated in a low-density detergent resistant membrane (DRM) fractions contributed to the expectation of their residence in lipid rafts (59).

Since then, accumulating evidence has improved the understanding of lipid rafts, also called membrane rafts, and rafts are currently viewed as fluctuating nanoscale assemblies enriched in sphingolipid, cholesterol, and proteins that can be stabilized to coalesce, forming platforms that function in membrane signaling and trafficking (60).

### Lipid raft function

The most important properties of lipid rafts are that they are small, dynamic, and heterogeneous, and can selectively recruit certain classes of proteins (61, 62). However, the underlying mechanism for the formation and functionality of lipid rafts has been largely unclear. Using single-molecule fluorescence tracking, Kusumi and colleagues recently found that GPI-anchored proteins formed dynamic, transient homodimer rafts in the plasma membrane, in a manner dependent on the interactions between their ectodomain protein portions (63). The homodimer formation seems to be the basic units for the organization and functions of membrane raft domains containing GPI-anchored proteins. Schütz and colleagues observed the relation between the physical state of the membrane domains and the partition of GPI-anchored proteins, and showed that GPI-anchored proteins do not reside in ordered domains (64). This report suggests that the phase partitioning is not a fundamental element of GPI-anchored protein organization in the plasma membrane, and also suggests the heterogeneity in the structure and function of membrane rafts.

Proteins with raft affinity include doubly acylated proteins such as Src family kinases, palmitoylated type-I transmembrane proteins, such as CD44 (65, 66), and receptor tyrosine kinases with two transmembrane subunits, such as inslulin receptor (67) and EGF receptor (68). Lipid rafts are implicated in many physiological cellular processes, such as protein membrane trafficking and signal transduction (62, 69). Cholesterol depletion is often used as a method for investigating the role of lipid rafts *in vitro*, although these studies are limited by non-specific effects. Nevertheless, these studies indicate that cholesterol is a crucial component of cell membranes that contributes to the organization of lipid rafts, and particularly to lipid rafts that contain large numbers of cancer-related signaling and adhesion molecules.

## Hyaluronan–CD44 Interaction and Lipid Rafts in Cancer

The dynamics of extracellular matrix production, degradation, and remodeling are carefully regulated during organ development; the dysregulation of extracellular matrix turnover and maintenance leads to abnormal cell behaviors and to failure of organ homeostasis and function, one of the most severe clinical outcomes in cancer (70). Altered cell adhesion and enhanced cell migration are the most prominent features of malignant tumor cells (71). The migratory properties of invasive tumor cells are affected by the interaction of their adhesion molecules with the surrounding extracellular matrix, and by growth factor signaling (72). The proteolytic cleavage and release (shedding) of membrane proteins’ ectodomain is a critical regulatory step in both physiological and pathological processes (73, 74). It was recently reported that oligomeric hyaluronan induce CD44 shedding from tumor cells (24).

Ectopic hyaluronan production is a frequent feature of colorectal, gastric, and breast cancers (75–77). Under normal physiological conditions, hyaluronan exists as a long polymer with a molecular weight of around 1 × 10^5^–1 × 10^7^ (1), whereas low-molecular weight hyaluronan is frequently detected in certain pathological conditions, such as inflammation (78) and cancer (79, 80), possibly due to the dysregulated expression of HASs and hyaluronidases. Hyaluronidase expression in prostate cancer tissues increased with tumor grade and metastasis, suggesting that prostate tumor cell-derived hyaluronidase might help the accumulation of low-molecular weight hyaluronan (80).

A prominent abnormality of certain malignant tumor cells, e.g., gliomas, is overexpression of the EGF receptor, and EGF induces CD44 shedding, that concomitantly enhance hyaluronan-mediated cell migration (81). PDGF and bradykinin also induce CD44 shedding, indicating that the Rho family of small GTPases plays crucial roles in the regulation of CD44 cleavage (81). TGF-β induces CD44 shedding in breast cancer cells (82), and this cleavage is MT1-MMP-dependent as previously described (83, 84). Granulocyte-colony stimulating factor (G-CSF) stimulates the MT1-MMP-mediated CD44 proteolysis in hematopoietic progenitor cells (85). Although the molecular mechanisms of the intracellular signaling in the tumor microenvironment that lead to CD44 shedding have been partially clarified (81, 86), the mechanism that triggers CD44’s shedding at the membrane is not understood.

There is growing interest in targeting lipid rafts for cancer prevention and treatment, because of their role in regulating various steps of cancer progression, including cancer cell migration and invasion (87), and because cancer-related proteins were listed in an unbiased proteomics analysis of these structures (88). Cell adhesion is a key factor in the metastatic spread of cancer cells, and regulating this process holds promise as an important therapeutic intervention for cancer. CD44 is the principal cell adhesion receptor expressed in cancer cells and implicated in cancer cell migration, invasion, and metastasis (89). Several reports recently demonstrated that CD44 is present in lipid rafts (90–100) (Table 2), and the role of lipid rafts in cancer cell adhesion and migration is being elucidated.

**Table 2:** **Hyaluronan-related proteins associated with lipid rafts**.

Protein	Cell type	Function in lipid rafts	Reference
CD44	Mammary adenocarcinoma	NHE1 activation	(93)
	Mammary adenocarcinoma	EGFR signaling	(94)
	Mammary adenocarcinoma	Cell migration	(95)
	Colon adenocarcinoma	Src-integrin signaling	(96)
	Glioblastoma	Cell migration	(97)
	Lung adenocarcinoma	Lamellipodia formation	(98)
	Lymphoma	Cell adhesion	(99)
	Myofibroblast	EGFR signaling	(100)
Hyal-2	Mammary adenocarcinoma	ECM degradation	(93)
	Mammary adenocarcinoma	N/A	(101)
HAS3	Mammary adenocarcinoma	Cell-surface protrusion	(102)
TLR4	Monocytic cell line	Cellular activation	(103)

*ECM, extracellular matrix; EGFR, epidermal growth factor receptor; HAS3, hyaluronan synthase 3; Hyal-2, hyaluronidase-2; NHE-1, Na^+^-H^+^ exchanger 1; TLR4, toll-like receptor 4*.

Lipid rafts play a pivotal role in CD44’s localization and function (97). Cholesterol depletion form human glioma cells using methyl-β-cyclodextrin (MβCD), an agent frequently used to disrupt lipid rafts, results in increased CD44 shedding, which was mediated by a transmembrane protease a disintegrin and metalloproteinase 10 (ADAM10). The CD44 shedding induced by cholesterol depletion is also seen in other tumor cells, such as pancreatic cancer cells. CD44 shedding can also be induced by a polyene macrolide antibiotic filipin that binds cholesterol and disperses it in the membrane, thereby disrupting lipid rafts by a different mechanism from MβCD. The cholesterol-lowering medication simvastatin also enhances CD44 shedding; it also blocks the stimulation of glioma cell migration by oligomeric hyaluronan or EGF. Taken together, these results suggest that cholesterol-lowering causes disordered CD44 localization, raft-dependent CD44 shedding, and the suppression of tumor cell migration that is dependent on hyaluronan’s size. CD44’s affiliation with lipid rafts is likely to occur through its palmitoylation, which may play a role in breast cancer malignancy (95).

In addition to CD44, several hyaluronan-related proteins, Hyal-2, HAS3, and toll-like receptor 4 (TLR4), have been reported to be associated with lipid rafts in cell membranes (93, 101–103) (Table 2). These membrane proteins are also likely to be involved in the regulation of lipid raft-associated interactions between hyaluronan and CD44. In addition, CD147 was found to regulate the lipid raft-associated CD44 function in cancer cell invasion (94, 104).

## Potential Roles of Hyaluronan–CD44 Interactions in Inflammation

The recruitment of lymphocytes from circulating blood to inflammatory sites or secondary lymphoid organs involves complementary receptor–ligand interactions between the lymphocytes and vascular endothelial cells. A multistep series of sequential receptor engagements enables the lymphocytes’ recognition of the endothelial surface and their subsequent extravasation (105). This process begins with the establishment of transient adhesive interactions that result in the rolling of lymphocytes along the endothelium under blood flow, and rolling is mediated by interactions between CD44 and hyaluronan (106, 107). The CD44–hyaluronan interaction is required for the extravasation of activated T cells from circulating blood to inflammatory sites (108). There is also evidence that the hyaluronan-binding ability of CD44 is correlated with the suppressor activity of CD4^+^CD25^+^ regulatory T cells (109).

CD44 does not bind hyaluronan constitutively in most immune cells, although CD44 is the principal receptor for hyaluronan in immune cells (89). Considering the ubiquitous distribution of CD44 and hyaluronan, tight regulation of the hyaluronan-binding ability of CD44 is likely to play a critical role in immunological responses: CD44 on resting T cells does not bind hyaluronan, but can be induced to bind it when the T cell is activated by antigen via the T-cell receptor (108–112). Various post-translational modifications on CD44, including glycosylation (113–115), chondroitin sulfate addition (116, 117), and sulfation (118, 119), are reported to affect its hyaluronan-binding ability. However, the membrane-based regulation of CD44’s hyaluronan-binding ability has not been clarified. A recent study demonstrated that the hyaluronan-binding ability of CD44 in T cells is upregulated by membrane cholesterol depletion, which causes CD44 to be dispersed from lipid rafts, although the effect is small (120). Cholesterol depletion also enhances the frequency of rolling adhesion under physiological flow conditions, suggesting that the CD44’s ligand-binding ability is governed by its cholesterol-dependent localization to lipid rafts.

## Perspectives

Epidermal growth factor receptor is one of the first reported growth factor receptors that exhibit raft affinity, and EGF induced the coalescence of EGF receptor-containing rafts with different type of lipid rafts that contain GPI-anchored proteins (68). This coalescence of different types of nanoscale assemblies possibly leads to the formation of functional platforms for transmembrane signaling and the initiation of the internalization of EGF receptors. In the case of hyaluronan receptor CD44, the function of hyaluronan in the regulation of lipid rafts may be in a similar way as proposed for EGF. As oligomeric hyaluronan can displace large hyaluronan from cells (4), it may modulate the raft coalescence that leads to form signaling platforms toward inflammation and cancer progression. Competitive binding assay showed that the minimum length of hyaluronan that can compete large hyaluronan binding to CD44 is 6-mer, and the nuclear magnetic resonance spectroscopy confirmed that 6-mer is the shortest oligomeric hyaluronan to give essentially full perturbation of CD44’s spectra (121). The structure of CD44’s hyaluronan-binding domain (HABD) solved by X-ray crystallography revealed that CD44 forms two different conformations upon binding to hyaluronan (122). To understand the molecular mechanism and associated energetics underlying the hyaluronan–CD44 binding interaction, Guvench group performed extensive all-atom explicit-solvent molecular dynamics (MD) simulations employing the adaptive biasing force free-energy methodology (123). They determined a clear description for the conformation-dependent affinity switching of the hyaluronan–CD44 interactions by MD simulation. These results should help the development of novel small compounds to therapeutics in inflammation and cancer by modulating hyaluronan–CD44 interactions, which may regulate the functionality of lipid rafts.

There has been growing interest in lipid rafts, and the lipid raft is a potential novel target in inflammation and cancer therapy (66, 124). Targeting hyaluronan–CD44 axis is one of the principal ways, and the lipid raft-targeted delivery of hyaluronan-grafted liposomes could have important applications in cancer therapy (125, 126). The modulation of CD44’s partition to lipid rafts may also offer potential avenues in inflammation and cancer therapy. Thus, the regulation and manipulation of hyaluronan–CD44 interactions through lipid rafts have potential applications for the prevention of inflammatory disorders and cancer.

## Conflict of Interest Statement

The author declares that the research was conducted in the absence of any commercial or financial relationships that could be construed as a potential conflict of interest.
